# Integrating metabolomics and microbiome analysis to unravel the plum blossom signature of premium Jiuqu Hongmei tea

**DOI:** 10.1016/j.fochx.2026.104072

**Published:** 2026-06-08

**Authors:** Jiang Cao, Yiwei Jin, Lei Zhang

**Affiliations:** a Innovative Drug Research Center, College of Life Sciences and Medicine, Key Laboratory of Plant Secondary Metabolism and Regulation of Zhejiang Province, Zhejiang Sci-Tech University, Hangzhou 310018, China; b Department of Pharmaceutical Botany, School of Pharmacy, Naval Medical University, Shanghai 200433, China

**Keywords:** Jiuqu Hongmei tea, Volatile compounds, Non-volatile compounds, Microorganisms

## Abstract

Jiuqu Hongmei (JQHM), a Chinese tea, features plum blossom aroma. However, its quality is often compromised by raw material and processing variability, and the chemical and microbial basis of this aroma remains unclear. To address this gap, integrated multi-omics assessed JQHM across grades. High grades showed enriched beneficial microbiota and critical aroma compounds, which synergistically formed a floral-fruity profile. Conversely, lower grades exhibited microbial dysbiosis and elevated theabrownins linked to sensory deterioration. Correlation networks revealed microbial regulation of metabolic pathways, deciphering the microbial-metabolite nexus governing the plum blossom signature and moving beyond empirical observations. By integrating metabolomics and microbiome analysis, this study uniquely demonstrates that the synergistic enrichment of specific beneficial taxa directly orchestrates the biosynthesis of key aroma compounds (linalool, geraniol, and methyl salicylate). This multi-omics strategy provides a mechanistic blueprint for precision quality control in JQHM production and establishes a paradigm for dissecting flavor formation in fermented foods.

## Highlights

• JQHM tea features a distinctive plum blossom flavor profile.
• The plum blossom character blends floral-fruity aroma with a smooth taste.
• Linalool, geraniol, and methyl salicylate are key to the plum blossom aroma.
• Premium grades are characterized by beneficial microbes.
• Multi-omics network revealed microbial regulation of aroma compound formation.

## Graphical abstract

**Figure:**
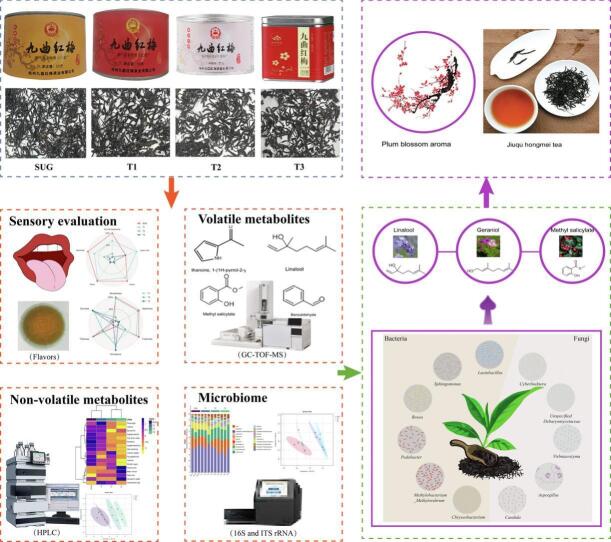

## 1 Introduction

Jiuqu Hongmei (JQHM), a distinct category of Chinese Gongfu black tea, is primarily cultivated in Hangzhou City, Zhejiang Province, China (alternatively termed Jiuqu Oolong). It is characterized by its thin, tightly curled leaves resembling fish hooks, a bright red liquor, a sweet and mellow taste with refreshing notes, and a signature plum-blossom-like aroma (Peng et al., 2023). The manufacturing process of JQHM follows the general stages of Gongfu black tea production, involving the steps of withering, rolling, fermentation, and drying of fresh tea leaves. Although it shares the full-fermentation pathway common to other types of black teas, it differentiates itself from other varieties through the use of local small-leaf tea materials and refined processing techniques (Li et al., 2024). However, the market quality of JQHM is often inconsistent due to variations in raw materials, processing conditions, and environmental factors. Among its key quality attributes, the distinctive plum blossom aroma serves as a critical marker for evaluating premium grades. Nevertheless, a comprehensive understanding of the sensory profile of plum blossom aroma and the key compounds responsible for its characteristic scent has not fully elucidated.

The overall flavor quality of tea is governed by two fundamental aspects: taste and aroma. These sensory attributes represent complex perceptual phenomena whose perception is not a simple sum of chemical constituents but rather an integrated experience arising from dynamic interactions, including enhancement, suppression, and transformation among multiple components (Wang et al., 2021). Specifically, the unique flavor characteristics of tea arise from volatile and non-volatile metabolites. By correlating these components with sensory attributes, key chemical constituents (e.g., polyphenols, caffeine, amino acids, and volatile compounds) that mediate flavor development have been identified (Jia et al., 2023). Therefore, variations in the content of sensory-related compounds are also recognized as a key driver of differences in tea flavor grades. In general, high-grade teas possess a greater abundance of volatile compounds compared to lower-grade teas. However, systematic characterization of aroma profiles in grade-standardized JQHM samples remains notably lacking. A comprehensive comparison of non-volatile and volatile compounds across different grades of JQHM would help clarify the chemical basis of its distinctive plum blossom scent. Such research would provide a scientific foundation for quality control and support the development of standardized evaluation systems for this tea.

The application of high-throughput sequencing has progressively revealed the composition and dynamics of microbial communities in post-fermented teas, highlighting their strong correlations with the formation of tea flavor profiles (Wang, Jia, et al., 2024; Zhang, Cai, et al., 2024). These microbial communities normally originate from two primary sources: the colonization by exogenous environmental microorganisms and the involvement of endophytic taxa inherent to the tea plant itself (Yao et al., 2024). For instance, the tea plant endophyte *Luteibacter* spp. has been implicated in the modulation of theaflavin biosynthesis, a microbially-driven process that contributes to the development of a fresher flavor profile in tea (Sun et al., 2019). Moreover, research on Fuzhuan brick tea production has identified six core functional microbes (e.g., *Aspergillus*, *Candida*, *Bispora*, *Penicillium*, *Unclassified_k_Fungi*, and *Unclassified_o_Saccharomycetales*) that are directly linked to the production of key volatile metabolites (Li et al., 2020). Variations in tea-associated microbial assemblies arise from raw material heterogeneity, processing conditions, and environmental factors. The initial microbial community structure is primarily determined by the quality and type of fresh tea leaves. For instance, fresh leaf grade (a critical determinant of black tea aroma) has been empirically and practically linked to distinct aromatic profiles in different grades of black tea, underscoring the significant role of raw material quality in shaping both the microbial community and the sensory characteristics of the final product (Wang et al., 2023). Beyond food fermentation, the profound impact of microbial community variations on host outcomes is evident in human health; for instance, a decrease in the gut bacterium *Flavonifractor plautii* and its metabolite phytosphingosine has been mechanistically linked to an increased susceptibility to metabolic disorders in individuals with a phlegm-dampness constitution (Li et al., 2025). Furthermore, processing stages dynamically regulate microbial community succession through key operations such as withering, rolling, and fermentation, thereby generating characteristic processing fingerprints (Jia et al., 2022). Collectively, these microbial variations act as both key biological drivers of flavor development and critical entry points for elucidating the microbial mechanisms underlying tea quality formation (Lang et al., 2025).

While previous studies have characterized the chemical profile or microbial succession of teas independently, a critical gap remains in linking specific microbial metabolic functions to the biosynthesis of signature aroma compounds (Wang et al., 2023; Jia et al., 2022). This gap is significant because the industrial production of JQHM suffers from severe batch-to-batch quality inconsistency, where the desired plum blossom trait is often lost due to undefined microbial activities. Single-omics approaches often fail to capture the complex, multi-kingdom interactions that dictate sensory quality (Peng et al., 2023). To bridge this gap, this study employs an integrated multi-omics strategy to move beyond mere correlation towards a mechanistic understanding of the plum blossom phenotype (Cai et al., 2022). Sensory evaluation was first conducted to define the distinct flavor profiles associated with each grade, enabling a scientifically-grounded characterization of the plum blossom aroma notes. High-performance liquid chromatography (HPLC) and gas chromatography-time-of-flight mass spectrometry (GC-TOF-MS) were further utilized to quantify dynamic changes in volatile and non-volatile compounds during distinct grade. Additionally, amplicon sequencing was applied to track shifts in microbial communities. By integrating multi-omics data, correlation networks were constructed to link sensory attributes with flavor compounds and to associate flavor compounds with microbial taxa. This approach elucidated the dynamic relationships between sensory profiles, microbial community structures, and the regulatory role of microbes in the formation of key aroma compounds across different tea grades. Overall, the findings in this study could provide theoretical insights into the plum blossom-like aroma of JQHM, bridge existing research gaps through multi-omics integration, and establish a scientific basis for precise quality control and the standardization of tea production practices.

## 2 Materials and methods

### 2.1 Chemicals

All chemicals and standards were obtained from reputable suppliers. High-purity (≥ 98%) total catechins (TC), gallic acid (GA), catechin (C), epicatechin (EC), epigallocatechin (EGC), epicatechin gallate (ECG), gallocatechin gallate (GCG), epigallocatechin gallate (EGCG), catechin gallate (CG), and caffeine (CAF) standards were purchased from Beijing Solarbio Science & Technology Co., Ltd. (Beijing, China). Isoquercitrin and neochlorogenic acid reference standards (purity >98%) were supplied by Chengdu Refines Biotechnology Co., Ltd. (Chengdu, China). Acetonitrile and methanol of HPLC grade were acquired from Sigma-Aldrich (St. Louis, MO, USA). Conventional chemical reagents including phosphoric acid and ethanol (analytical grade) were provided by Sinopharm Chemical Reagent Co., Ltd. (Shanghai, China). 2-Octanol (internal standard, purity ≥99%) and n-alkane standard solution (C7 - C30) were obtained from Sigma-Aldrich (Shanghai, China). Ultrapure water (resistivity ≥18.2 MΩ·cm) was prepared using a Smart Ultra-pure Water System (Heal Force, China).

### 2.2 JQHM tea samples

In this study, JQHM tea samples of different quality grades were purchased from Shuangpu Town, Xihu District, Hangzhou, Zhejiang Province, China, provided by the Hangzhou Longjing Tea Industry Group Co., Ltd. The teas were classified into four grades: Super Grade (SuG), First Grade (T1), Second Grade (T2), and Third Grade (T3). All samples were ground manually using a liquid nitrogen-pre-cooled mortar and passed through an 80-mesh sieve. The powdered teas were then packaged in tinfoil bags and stored at 4 °C until analysis.

### 2.3 Sensory evaluation

The tea infusion was prepared following the national standard GB/T 23776-2018 (Methodology for Sensory Evaluation of Tea). Briefly, 3.0 g of tea leaves were accurately weighed into a standard assessment cup and subjected to three consecutive infusions using freshly boiled water. The infusion times were rigorously set at 2, 3, 5, 10, and 60 min, respectively. The infusion at 5 min (the third infusion) served as the primary basis for overall quality judgment, while the other infusions provided supplementary information. Sensory evaluation was performed by a trained panel consisting of 8 panelists from Zhejiang Sci-Tech University. Prior to the formal assessment, all panelists underwent a rigorous training session focused on identifying the specific sensory attributes of JQHM tea (including the plum blossom aroma) until a consensus on the scoring criteria was reached. With reference to the method described by Wu et al. with slight modifications, panelists scored key attributes, including dry leaf appearance, liquor color, aroma, taste, and infused leaf appearance (Wu et al., 2024). The intensity of each sensory attribute was rated on a 0–10 scale, where 0 = absent/imperceptible, 3 = weak, 5 = moderate, 7 = strong, and 10 = very strong intensity. The final sensory score for each attribute was calculated as the arithmetic mean of the scores provided by the 8 panelists. To ensure reliability and reproducibility, the sensory assessment for each tea sample was performed in triplicate.

The sensory evaluation was conducted in accordance with the guidelines of Zhejiang Sci-Tech University. In compliance with institutional policy, formal ethical approval was exempted for this low-risk food tasting study involving non-invasive procedures. Written informed consent was obtained from all participants prior to the study. All participant information was anonymized to protect privacy and confidentiality in accordance with relevant legal standards.

### 2.4 Measurement of non-volatile components in samples

To comprehensively profile the non-volatile metabolome, we employed both spectrophotometric and chromatographic techniques. The contents of water extract (WE), total tea polyphenols (TPs), free amino acids (AA), and soluble sugars (SS) were determined using standard spectrophotometric methods according to national standards. Specifically, WE was measured according to GB/T 8305-2013; AA content was determined using the ninhydrin colorimetric method (GB/T 8314-2013); TPs were analyzed following GB/T 8313-2018; total flavonoids were quantified by aluminum trichloride colorimetry; and caffeine (CAF) content was determined in accordance with GB 5009.139-2014.

Crucially, to meet the requirements for precise metabolite identification, High-Performance Liquid Chromatography (HPLC) was utilized for the targeted analysis of specific bioactive compounds. Individual catechins (C, EC, EGC, ECG, GCG, EGCG, CG), total catechins (TC), and gallic acid (GA) were quantitatively analyzed using HPLC as previously described (Jia et al., 2022).

### 2.5 Measurement of volatile components in samples

Given the distinct physicochemical properties of aroma compounds, volatile metabolites were analyzed using Headspace Solid-Phase Microextraction combined with Gas Chromatography-Time of Flight Mass Spectrometry (HS-SPME-GC-TOF-MS) as previously described (Hao et al., 2023). Take 800 ± 5 mg sample into the 20 mL headspace bottle, add 10 μL of 2-Octanol (10 mg/L stock in dH_2_O) as internal standard. In SPME cycle of PAL rail system. Incubate temperature is 60 °C; Preheat time in 15 min; Incubate time in 30 min; Desorption time is 4 min.

GC–MS analysis was performed using an Agilent 7890 gas chromatograph system coupled with a 5977B mass spectrometer. The system utilized a DB-Wax. Injected in Splitless Mode. Helium was used as the carrier gas, the front inlet purge flow was 3 mL min^−1^, and the gas flow rate through the column was 1 mL min^−1^. The initial temperature was kept at 40 °C for 4 min, then raised to 245 °C at a rate of 5 °C min^−1^, kept for 5 min. The injection, transfer line, ion source and quad temperatures were 250, 250, 230 and 150 °C, respectively. The energy was −70 eV in electron impact mode. The mass spectrometry data were acquired in scan mode with the *m*/*z* range of 20–400, solvent delay of 2.37 min.

### 2.6 Calculation of odor activity value (OAV)

OAVs were applied as a key metric to evaluate the contribution of volatile compounds to the overall aroma profile of the tea samples. It is calculated as the ratio of the concentration of a compound (C) to its odor threshold (OT) in water (OAV = C/OT). Volatile compounds with an OAV greater than 1 are generally considered to make a significant contribution to the aroma characteristics of the sample (Wang et al., 2020).

### 2.7 DNA extraction and amplification sequencing

The microbial community analysis of tea samples in this study was primarily conducted following established standard methods (Yuan et al., 2024). The general procedure was as follows: tea samples were first ground in liquid nitrogen, then homogenized in sterile saline and subjected to constant-temperature oscillation to extract microorganisms (Zhang, Wang, et al., 2024). Microbial cells were collected by centrifugation, and total DNA was extracted using the OMEGA Soil DNA Kit. After passing quality control checks, the DNA was used for subsequent analysis. PCR amplification was performed targeting the hypervariable V4–V5 regions of the bacterial 16S rRNA gene using the primer pair 515F (5′-GTGCCAGCMGCCGCGGTAA-3′) and 907R (5’-CCGTCAATTCMTTTRAGTTT-3′). For fungal communities, the ITS1 region was targeted using the primer pair ITS5F (5’-GGAAGTAAAAGTCGTAACAAGG-3′) and ITS2R (5′-GCTGCGTTCTTCATCGATGC-3′). These regions were selected because the V4–V5 region offers superior phylogenetic resolution for discriminating closely related bacterial species in fermented foods, and ITS1 is widely recognized as the most informative region for identifying diverse fungal taxa. The amplified products were purified and then sequenced on the Illumina MiSeq platform for high-throughput sequencing. The raw sequencing data were processed using the QIIME2 (v2023.9) pipeline. The workflow included demultiplexing, primer trimming, and denoising based on the DADA2 algorithm (v1.20) to generate Amplicon Sequence Variants (ASVs). Key parameters were optimized for read quality: forward and reverse reads were truncated at 250 bp and 200 bp, respectively, to remove low-quality base calls. Taxonomic annotation of the ASVs was finally performed using the SILVA database (v138). A confidence threshold of 0.7 was applied during the taxonomic classification to ensure the accuracy of species assignment.

### 2.8 Statistical analysis

All data are expressed as mean ± SEM. Statistical analyses were conducted using GraphPad Prism (v10.1.2), with between-group differences assessed by a two-tailed unpaired *t*-test and multi-group comparisons analyzed via one-way ANOVA followed by Dunnett's post hoc test. The same ANOVA method was applied to alpha diversity indices in microbial analysis, considering *P <* 0.05 statistically significant. Chromatography raw data were processed using LECO Chroma TOF 4.3× software and the NIST database for baseline correction, peak alignment, deconvolution, and integration. Spearman correlations among sensory attributes, chemical compounds, and microbial communities were computed in RStudio (v4.2.2) with the “psych” package, where *P <* 0.05 and *|r|* > 0.7 defined significant correlations (positive if *r* > 0.7, negative if *r* < −0.7). Correlation networks were visualized using Gephi (v0.9.4).

## 3 Results and discussion

### 3.1 Sensory characteristics of different grades of JQHM tea

**Fig. 1:**
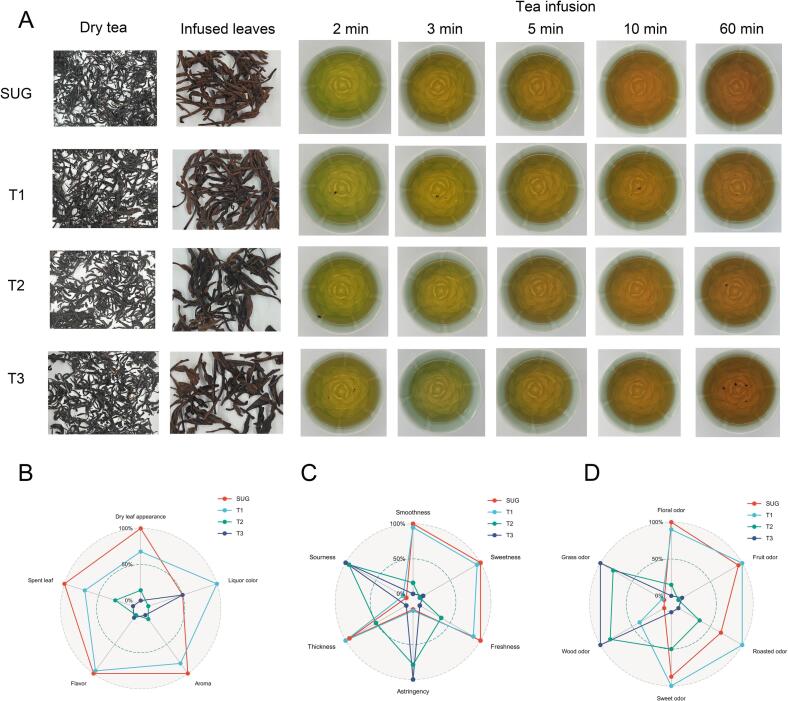
Sensory characteristics of different grades of JQHM tea. (A) The appearance of the dry tea, infused leaves and the tea infusion. (B) Sensory evaluation radargram of different grades of JQHM black tea. (C) Aroma radargram of different grades of JQHM tea. (D) Taste radargram of different grades of JQHM tea.

JQHM tea is highly appreciated for its well-layered flavor profile, particularly its distinctive sweet and cooling aroma reminiscent of plum blossoms, which greatly contributes to its consumer appeal. The dry leaf appearance, spent leaves, and liquor color were initially evaluated in this study, revealing notable differences across commercial grades (Fig. 1A). One classic visual characteristic of this tea is its tightly curled leaf form. In this study, higher grades exhibited finer, more compact, and uniform strands, corresponding to superior visual ratings. In addition, although all tea infusions showed an orange-red color, those from higher grades displayed a brighter and more transparent liquor. Subsequent systematic sensory analysis, conducted in accordance with the national standard of methodology for sensory evaluation of tea (GB/T 23776–2018), showed that the overall sensory scores of SUG and T1 grades were significantly higher than those of T2 and T3. This result confirms their classification as premium products, aligning with previous findings (Peng et al., 2023). Moreover, the observed inverse correlation between grade and sensory scores for aroma and flavor establishes them as key discriminators of JQHM tea quality.

To further elucidate the flavor differences among various grades of JQHM tea, a descriptive analysis was undertaken using a predefined sensory lexicon to score specific taste and aroma attributes (Yuan et al., 2024). The sensory evaluation indicated that high-grade teas (SUG, T1) received significantly higher scores for key taste attributes (Smoothness, Sweetness, Freshness) and aroma attributes (Floral, Fruity) compared to lower grades (T2, T3). Conversely, the lower-grade teas were characterized by more pronounced Sourness, Astringency, and Woody and Grassy notes. Thus, the esteemed plum-blossom character of high-grade JQHM corresponds to a specific sensory signature defined by a floral-fruity aroma and a smooth, sweet, fresh taste. In summary, this study demonstrated clear sensory distinctions between the various grades of JQHM tea.

### 3.2 Analysis of non-volatile compounds in different grades of tea

**Fig. 2:**
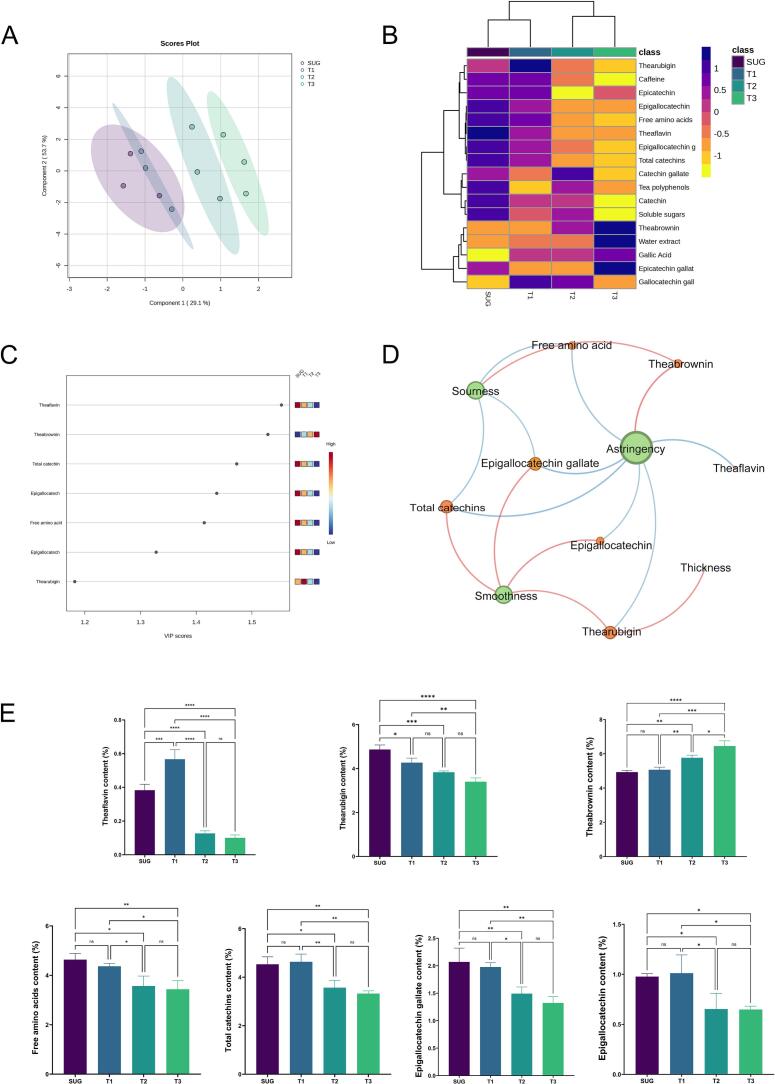
Results of multivariate statistical analysis of non-volatile compounds in JQHM tea. (A) Partial Least Squares Discriminant Analysis. (B) Heat map of clustering of characteristic non-volatile compounds. (C) VIP values of non-volatile compounds. (D) Correlation analysis of taste metabolites, and tasting results of JQHM teas from different grades. The red line connects the two positively correlated metabolites and the blue line connects the two negatively correlated metabolites. The thickness of the line indicates the strength of the correlation. The thicker the line, the stronger the correlation. (E) Changes in tea quality-related metabolites during the processing of black tea. (*n* = 3, ns *P >* 0.05, * *P <* 0.05, ** *P <* 0.01, *** *P <* 0.001, **** *P <* 0.0001). (For interpretation of the references to color in this figure legend, the reader is referred to the web version of this article.)

To further investigate the factors underlying the distinct “plum-blossom” flavor profiles among different grades of JQHM tea, the contents of several key non-volatile compounds were quantified. The detected non-volatile compounds were initially subjected to partial least squares-discriminant analysis (PLS-DA) and hierarchical clustering analysis (HCA) (Fig. 2A & B). The integrated results demonstrated that the four grades of JQHM tea could be clearly classified into two clusters, consistent with sensory evaluation results. The premium grades SUG and T1 exhibited overlapping distributions in the PLS-DA score plot and were grouped together in the HCA, indicating their similar non-volatile compositional profiles. These results suggested that non-volatile compounds serve as critical indicators for grade discrimination and contribute significantly to the “plum-blossom” flavor.

Using a criterion of variable importance in projection (VIP) > 1, seven key non-volatile compounds (AA, TFs, TRs, TBs, TC, GCG, and ECG) were further discriminated as signature components (Fig. 2C). Among these, the contents of all compounds except TBs decreased with declining tea grade (Fig. 2E). Inversely, the abundance of TBs was significantly elevated in lower-grade samples relative to the premium group (*P <* 0.05, Fig. 2E). This divergent trend can likely be explained by the underlying differences in the quality of raw materials and the applied processing techniques. As reported in the literature, the superior quality of high-grade teas is closely linked to the use of tender spring shoots, whose active nitrogen metabolism leads to the accumulation of high concentrations of amino acids and polyphenols (Tang et al., 2023). This chemical profile offers an ideal precursor pool for theaflavin synthesis. In addition, the well-regulated fermentation process and precisely timed drying step employed in high-grade tea production are essential for preserving TFs and TRs, thus limiting the formation of TBs (Cai et al., 2023; Chen et al., 2024). In contrast, lower-grade teas are typically manufactured from coarser leaves characterized by a carbon-metabolism-dominated physiological state. This results in increased fiber content, diminished free amino acid levels, and a pronounced tendency for TBs accumulation, Lower grades (T2, T3) exhibited significantly elevated levels of TBs, which correlated with darker infusions and astringent tastes. While generally attributed to over-fermentation, the underlying mechanism involves specific microbial dysregulation. Under suboptimal processing conditions (e.g., excessive moisture or temperature), the proliferation of certain bacteria (e.g., *Bacillus*, *Clostridium*) and filamentous fungi accelerates the oxidation and condensation of catechins. These microbes secrete high levels of extracellular enzymes, such as polyphenol oxidases and peroxidases, which catalyze the rapid polymerization of flavanols into high-molecular-weight TBs. This microbial-driven enzymatic cascade explains the loss of brightness and the increase in harshness observed in lower-grade teas, primarily caused by over-fermentation resulting from suboptimal processing conditions (Xing et al., 2024).

Finally, correlation network analysis was performed to associate non-volatile compounds with sensory evaluations to clarify the impact of characteristic non-volatile compounds on sensory attributes (Fig. 2D). Sourness and astringency showed significant positive correlations with TBs content (*P <* 0.05, *r* > 0.7). Previous studies have demonstrated that TBs, primarily formed during over-fermentation, impart undesirable sensory characteristics such as dull liquor color, a bland taste, and coarse astringency, which are generally associated with impaired tea quality (Lu et al., 2024). Thus, elevated levels of TBs in lower-grade teas contribute to the intensification of sour and astringent sensations. In contrast, both sourness and astringency are negatively correlated with AA content. Existing studies have reported that AA content is strongly associated with the quality of both green and black teas (Jia et al., 2022). AAs have been found to mitigate the astringency of TPs and the bitterness of CAF, while also imparting refreshing aromas and contributing to a sense of mildness (Lin et al., 2014). Furthermore, high-grade teas showed significant positive correlations between smoothness and the combined levels of TFs, TC, EGCG, and EGC. The presence of TRs, along with total catechins, EGCG, and EGC, synergistically enhances the smoothness of high-grade black tea. TFs and TRs are among the most critical quality indicators formed during fermentation, influencing both the brightness and redness of the tea infusion (Chiang et al., 2022). It has been reported that the molecular structure of TRs can form stable colloidal systems with catechins (particularly EGCG and EGC) via hydrogen bonding and hydrophobic interactions (Long et al., 2023). This colloidal system not only enhances the thickness and fullness of the tea infusion but also masks the irritating phenolic groups of catechins, thereby improving the oral tactile sensation. In summary, the heightened sourness and astringency in lower-grade teas can largely be attributed to elevated levels of TBs. In contrast, the well-balanced combination of AA, TRs, TBs, TC, GCG, and ECG collectively contributes to the smooth and mellow taste profile characteristic of high-grade JQHM tea.

### 3.3 Analysis of volatile compounds in different grades of tea

**Fig. 3:**
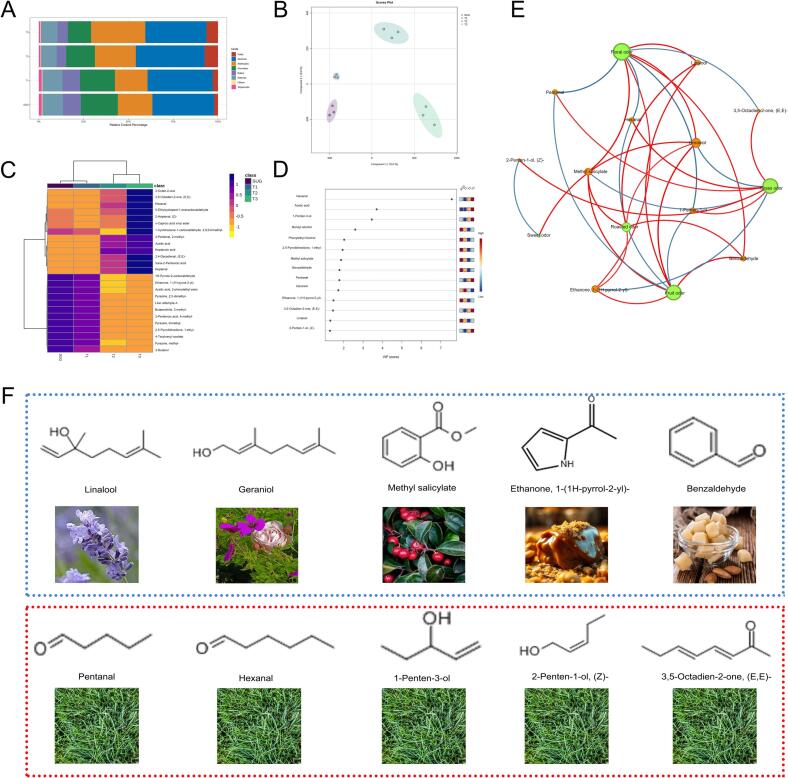
Results of multivariate statistical analyses of volatile compounds in JQHM tea. (A) Relative content percentage stacking plot. (B) Partial Least Squares Discriminant Analysis. (C) Heat map of clustering of characteristic volatile compounds. (D) VIP *>* 1 for characteristic volatile compounds. Characteristic volatile compounds. (E) Characteristic volatile compounds and aroma sensory correlation network. The red line indicating a positive correlation and the blue line a negative correlation. The thickness of the lines indicates the strength of the correlation, and the size of the nodes indicates the degree of connectivity. (F) The aroma sketch map of the 10 volatile compounds. (For interpretation of the references to color in this figure legend, the reader is referred to the web version of this article.)

To elucidate the mechanisms responsible for flavor variations among different grades of JQHM tea, volatile compound profiling was conducted using GC-TOF-MS. A total of 129 volatile compounds were identified and classified into aldehydes, ketones, alcohols, esters, terpenoids, aromatic compounds, acids, and other categories. Aldehydes, ketones, and alcohols were found to be the dominant categories across all grades, collectively accounting for more than 70% of the total volatile content (Fig. 3A). Consistent with the approach applied to non-volatile compounds, sample distinctions were evaluated using PLS-DA and HCA (Fig. 3B & C). The PLS-DA analysis revealed distinct clustering among the four tea grades, indicating significant compositional differences. Samples of higher grades (SUG and T1) clustered more closely in both dimensions, correlating with their similar sensory attributes such as floral, fruity, and sweet notes. In addition, cluster analysis based on 25 major aroma compounds further separated the samples into a premium group (SUG and T1) and a lower-grade group (T2 and T3).

VIP analysis was also applied to assess the contribution of each volatile compound to group separation. Fourteen aroma compounds with VIP scores exceeding 1 were identified as key discriminators (Fig. 3D). Several compounds, including 1-(1H-pyrrol-2-yl)-ethanone, benzyl alcohol, linalool, geraniol, methyl salicylate, phenethyl alcohol, benzaldehyde, and 1-ethyl-2,5-pyrrolidinedione, were present at significantly higher levels in high-grade teas, indicating their role as characteristic aroma compounds of premium grades (*P* < 0.05, Fig. 3D). In contrast, lower-grade teas were enriched with compounds such as (E, E)-3,5-octadien-2-one, 1-penten-3-ol, pentanal, hexanal, (Z)-2-penten-1-ol, and acetic acid. Previous studies have supported that the association of these compounds with distinct flavor profiles (Chambers IV & Koppel, 2013). Thus, the unique plum-blossom-like aroma of high-grade JQHM tea is closely associated with the differential accumulation of these volatile compounds.

Not all differentially expressed volatile compounds in black tea actually contribute to aroma formation, as their impact depends on whether they reach their olfactory thresholds (Xiao et al., 2025). To elucidate the formation mechanism of the plum blossom-like aroma in JQHM tea, this study calculated the OAVs of the differential volatile compounds. An OAV ≥ 1 indicates that a compound contributes to the overall aroma, with higher values indicating greater importance (Guo et al., 2022). As shown in Table 1, 10 out of 14 differential compounds had OAV ≥ 1, including linalool, geraniol, and methyl salicylate, which are considered key contributors to its unique aroma profile.

**Table 1:** OAV of differential aromatic compounds in JQHM black tea.

Compounds	Odor description^a^	Odor type	Ot^b^	SUG	T1	T2	T3
Linalool	Floral, lavender-like, slightly woody	Floral, Woody	6.0	77.6 ± 6.31	73.31 ± 6.56	46.44 ± 2.58	61.1 ± 1.26
Geraniol	Sweet, rose-like, floral	Floral, Rose	3.5	208.31 ± 10.65	210.34 ± 8.87	170.67 ± 23.58	164.1 ± 5.46
Methyl salicylate	Sweet, wintergreen-like	Minty, Sweet	40.0	10.89 ± 0.62	9.91 ± 0.88	3.65 ± 0.71	7.55 ± 0.37
Ethanone, 1-(1H-pyrrol-2-yl)-	Nutty, roasted, caramel-like	Nutty, Caramel	21.0	7.57 ± 0.18	6.61 ± 0.19	<1	1.33 ± 0.11
Benzaldehyde	Bitter almond, marzipan-like	Nutty, Almond	350.0	1.33 ± 0.05	1.11 ± 0.18	<1	<1
Benzyl alcohol	Faintly sweet, floral, slightly aromatic	Floral, Fruity	100,000.0	<1	<1	<1	<1
Phenylethyl Alcohol	Rosy, honey-like, floral	Floral, Honey	1100.0	<1	<1	<1	<1
Pentanal	Pungent, almond-like, malt-like	Pungent, Nutty	12.0	3.13 ± 0.24	2.97 ± 0.42	8.78 ± 1.96	18.47 ± 1.27
Hexanal	Green, grassy, fatty	Green, Grassy	4.5	31.81 ± 3.05	26.49 ± 1.08	88.98 ± 10.27	206.72 ± 13.34
1-Penten-3-ol	Pungent, grassy, slightly chemical	Green, Pungent	3.8	59.79 ± 0.65	54.32 ± 7.08	96.08 ± 12.43	151.55 ± 16.04
2-Penten-1-ol, (*Z*)-	Intense green, grassy, herbal	Green, Herbal	200.0	<1	<1	<1	1.31 ± 0.11
Acetic acid	Sour, vinegar-like	Sour, Vinegar	220,000.0	<1	<1	<1	<1
3,5-Octadien-2-one, (E,E)-	Fatty, green, earthy	Fatty, Green	1.0^4^	65.04 ± 5.14	61.3 ± 1.5	102.52 ± 13.63	219.97 ± 9.07
2,5-Pyrrolidinedione, 1-ethyl-	Faint caramel, creamy, sweet	Caramel, Creamy	50,000	<1	<1	<1	<1

a odor description found in the literature with database (Flavornet; https://pubchem.ncbi.nlm.nih.gov/).

b OT: odor threshold data were obtained from the Leibniz-LSB@TUM Odorant Database (https://www.leibniz-lsb.de/en/databases) and supplemented with newly measured data according to relevant literature and the reference book by Gemert. (Gemert, L. et al., 2011).

Further correlation network analysis was performed to associate compounds meeting both VIP *>* 1 and OAV > 1 criteria with sensory attributes. The results demonstrated strong positive correlations between floral/fruity notes and linalool, geraniol, and methyl salicylate (Fig. 3E), confirming their role as primary contributors to the plum-like flavor profile. Specifically, linalool contributes a fresh, lily-like aroma with subtle woody undertones, establishing a refined floral foundation. Complementing this, geraniol delivers a sweet, rosy scent that enhances the characteristic sweetness, while methyl salicylate introduces a wintergreen-like coolness, adding complexity. Quantitatively, this sensory profile is corroborated by distinct concentration gradients across grades (Table S1): linalool (278.6–465.6 μg/kg), geraniol (574.4–736.2 μg/kg), and methyl salicylate (145.9–435.7 μg/kg) were significantly higher in premium grades (SuG, T1) than in lower grades (T2, T3), directly underpinning the aroma intensity (Table S1). Thus, the synergistic interaction among these three compounds underpins the cool, subtly sweet aroma reminiscent of plum blossoms: linalool establishes a fresh foundation, geraniol provides the core sweetness, and methyl salicylate accentuates the delicate sweetness and cooling sensation. The harmonious blending of these aromatic layers effectively replicates the elegant, sweet, yet refreshing nuance distinctive of plum blossoms. In contrast, the concentration of aldehydes (e.g., pentanal, hexanal), which are more abundant in lower-grade teas, showed a significant positive correlation with grassy aroma attributes. These aldehydes are derived from lipid oxidation during processing and exhibit pronounced green, grassy notes (Wang, Lei, et al., 2024). When accumulated to excessive levels without adequate conversion, they overwhelm the desirable floral and fruity aromas of black tea, leading to a course, astringent, and green-tasting profile that compromises overall quality.

### 3.4 Analysis of bacterial communities in different grades of tea

In this study, internal transcribed spacer (ITS) and 16S rRNA amplicon sequencing were employed to systematically analyze microbial communities in JQHM across different quality grades, aiming to elucidate the effects of microbial activities on its plum blossom-like aroma and to reveal differences in microbial diversity and community structure at the genus level. α-Diversity analysis, based on the Shannon index and observed operational taxonomic units (OTUs), was employed to assess microbial richness and evenness within the JQHM tea samples. Results showed significantly lower bacterial and fungal α-diversity in high-grade JQHM compared to low-grade samples (*P* < 0.05, Fig. S1A & S1B). High-quality black teas typically employ controlled environmental parameters (e.g., temperature, humidity) and extended processing times during withering and fermentation, which selectively enrich specific dominant microbial consortia rather than promoting generalized microbial proliferation (Gao et al., 2025). Concurrently, rigorous processing steps suppress or eliminate transient environmental microbes, resulting in a more concentrated and phylogenetically distinct final microbial profile (Wang, Xie, et al., 2024). Although this optimization reduces overall diversity, it facilitates the enrichment of specific functional microbes critical for driving the formation of high-grade tea-specific aroma compounds and stable sensory attributes. β-Diversity analysis further resolved microbial community structures across production regions. Bacteria and fungi from JQHM samples clustered distinctly into two groups: SUG and T1 shared similar bacterial community structures, whereas T2 and T3 formed a separate cluster (Fig. S1C & S1D). This clustering pattern aligned closely with prior sensory evaluation and flavor metabolite data, strongly supporting the role of microbial activities in modulating rose-like aroma development.

To further resolve microbial community structure, we analyzed the composition and relative abundance of bacteria and fungi in black tea at genus level. Results revealed that dominant bacterial genera (accounting for >60% of total bacteria) included *Sphingomonas*, *Muribaculaceae*, *Pseudomonas*, *unclassified Sphingomonadaceae*, *Methylobacterium_Methylorubrum*, *Helicobacter*, *Variovorax*, *Acinetobacter*, *Bacteroides*, and *Phyllobacterium* (Fig. S1E). Most of these genera have been previously detected in black tea products, with *Sphingomonas*, *Variovorax*, *and Methylobacterium_Methylorubrumidentified* as dominant in prior studies (Nurmilah et al., 2022). For fungi, dominant genera (comprising 77%–90% of total fungi) included *unclassified Eukaryota*, *unclassified Pleosporales*, *Unspecified Debaryomycetaceae*, *Unspecified Eudicotyledonae*, *Aspergillus*, and *Vishniacozyma* (Fig. S1F). Notably, *Unspecified Debaryomycetaceae*, *Aspergillus*, and *Vishniacozyma* have been identified by multiple studies as core functional fungi during the fermentation of dark tea.

Subsequently, LEfSe analysis (Linear Discriminant Analysis Effect Size) was performed to identify core differential genera across quality grades. Bacterial LEfSe results identified 18 significantly discriminative taxa, indicating unique bacterial signatures for each grade (Fig. S1G). The SUG group was enriched with *Chryseobacterium*, *Bosea*, *Methylobacterium_Methylorubrum*, and *Sphingomonas*. *Sphingomonas* and *Chryseobacterium*, which are gram-negative bacteria commonly found in diverse natural environments such as soil and water, can be introduced into the fermentation system through raw materials or the processing environment (Li et al., 2018). Research indicated that these microorganisms could catalyze the moderate degradation of polysaccharides and amino acids, thereby facilitating the formation of TFs and TRs, which are key pigments contributing significantly to the liquor's color intensity, brightness, and mouthfeel (Liu et al., 2023). The genus *Methylobacterium–Methylorubrum*, belonging to the family *Methylobacteriaceae*, has been identified as a core functional bacterium in tea fermentation (Li et al., 2018). Given that studies have reported this genus possesses the molecular machinery to synthesize key aroma compounds such as linalool, the significant positive correlation observed between its abundance and linalool concentration likely originates from its direct synthetic activity and/or its indirect influence on the metabolic regulatory network of the host plant (Yurimoto et al., 2021). In the T1 group, the relative abundances of *Pedobacter*, *Variovorax*, and *Lactobacillus* were significantly higher than those in other groups. Although the first two types of bacteria have been widely detected during black tea processing, their specific mechanisms of action in shaping tea quality remain incompletely understood. It is known that *Variovorax*, as a major endophytic bacterium in tea plants, plays an important role in regulating tea plant growth and development, while the lactic acid produced by *Lactobacillus* can moderate the bitterness of the tea infusion, thereby contributing to a more balanced taste profile (Cheng et al., 2025). Conversely, in low-grade tea groups (e.g., T2, T3), a significant increase in the relative abundance of several bacterial taxa has been observed, notably *Clostridia_UCG_014*, *Escherichia-Shigella*, *Acinetobacter*, *Brevundimonas*, and *Aquabacterium* (Fig. S1G). The presence and enrichment of these microorganisms, particularly strains indicative of animal intestinal origins, serve as a typical marker for fecal contamination during processing, reflecting inadequate sanitary measures and potential external contamination (Zheng et al., 2025). Furthermore, under inappropriate fermentation conditions, these microbial communities can produce off-flavor compounds. For instance, excessive *Clostridia_UCG_014* may yield butyric or valeric acids, which is associated with rancid notes (Szczuko et al., 2024). Thus, our results confirmed that the flavor characteristics of high-grade black tea are partially driven by the directed regulation of bacterial communities during fermentation.

Fungal LEfSe analysis revealed significant differences in microbial taxa composition across the four experimental groups. The T3 group exhibited the highest number of differentially abundant genera, indicating the most substantial microbial compositional divergence from the other groups, as reflected by β-diversity analysis (Fig. S1H). Specifically, the premium black tea groups were primarily enriched with a consortium of functional fungi. Among these, the genus *Aspergillus* was significantly correlated with the production of key volatile organic compounds (VOCs), contributing to the complex aroma profile characterized by floral, fruity, woody, and minty notes. This fungal genus employs a diverse enzymatic arsenal, including pectinases and cellulases for aroma precursor release, glycosyltransferases/glycosidases for taste modulation, and lipases for lipid-derived aroma generation, to synergistically shape the distinctive sensory profile of tea (Yang et al., 2023). Concurrently, several yeast genera known as aroma producers and environmental modulators in black tea, including *Candida*, *Vishniacozyma*, *Cyberlindnera*, and *unclassified Debaryomycetaceae*, were also detected in the premium tea groups. These yeasts contribute to the aromatic profile by synthesizing esters and alcohols, thereby enhancing the complexity of the tea's aroma. These yeasts are known to participate in the synthesis of esters and alcohols, further enriching the tea's aromatic complexity (Zou et al., 2025). Thus, the fungal community in premium black tea fermentation exhibits a clear functional division, with *Aspergillus* acting as the primary decomposer of complex substrates and diverse yeasts serving as specialized aroma producers. Their synergistic interaction forms a highly efficient microbiome essential for developing the characteristic flavor and quality of high-grade black tea. In contrast, the fungal profiles of the low-grade T2 and T3 groups were distinctively marked by an enrichment of known phytopathogenic and saprophytic genera. Specifically, T3 exhibited dominance by *Gibberella*, *Fusarium*, *Colletotrichum*, and *Diaporthe*, whereas T2 was primarily enriched with *Phaeosphaeria* and *Cladosporium*. Given that many of these taxa are well-established causes of agricultural diseases (e.g., head blight, leaf spots), their prominence suggests potential deficiencies in fermentation control or raw material quality (Huang et al., 2022). For instance, a previous research has demonstrated that infection by *Cladosporium* significantly reduces the content of key quality components in tea leaves, such as caffeine, theanine, and catechins, while also damaging leaf cell structure and chloroplast function, thereby directly impairing both the commercial value and sensory quality of the tea (Liu et al., 2024)*.* It is therefore hypothesized that these microorganisms may contribute to off-flavors or other quality defects in the final product, underscoring the critical importance of implementing stringent processing protocols to ensure premium tea quality.

In summary, the distinct flavor profiles of high-grade and low-grade teas are primarily driven by variations in the microbial community structure during fermentation. High-quality black tea typically features an enrichment of beneficial bacteria (e.g., *Methylobacterium–Methylorubrum*, *Sphingomonas*, *Lactobacillus*) and functional fungi such as *Aspergillus*, and yeasts like *unclassified Debaryomycetaceae*,. These microorganisms secrete specific extracellular enzymes that catalyze the oxidation of tea polyphenols into key flavor compounds like TFs, while concurrently synthesizing aromatic esters and alcohols, collectively contributing to a mellow taste, a bright red liquor color, and a complex aroma profile. Conversely, low-grade teas often display a dominance of plant pathogens or saprophytic microorganisms, which compete for and consume critical flavor precursors, and may produce off-flavor metabolites, thereby undermining the harmony and purity of the tea's sensory attributes. Therefore, precise regulation of the beneficial microbial community is crucial for ensuring and enhancing the overall quality of tea products.

### 3.5 Correlation analysis of the relative abundance of bacterial community and tea quality-related metabolites

**Fig. 4:**
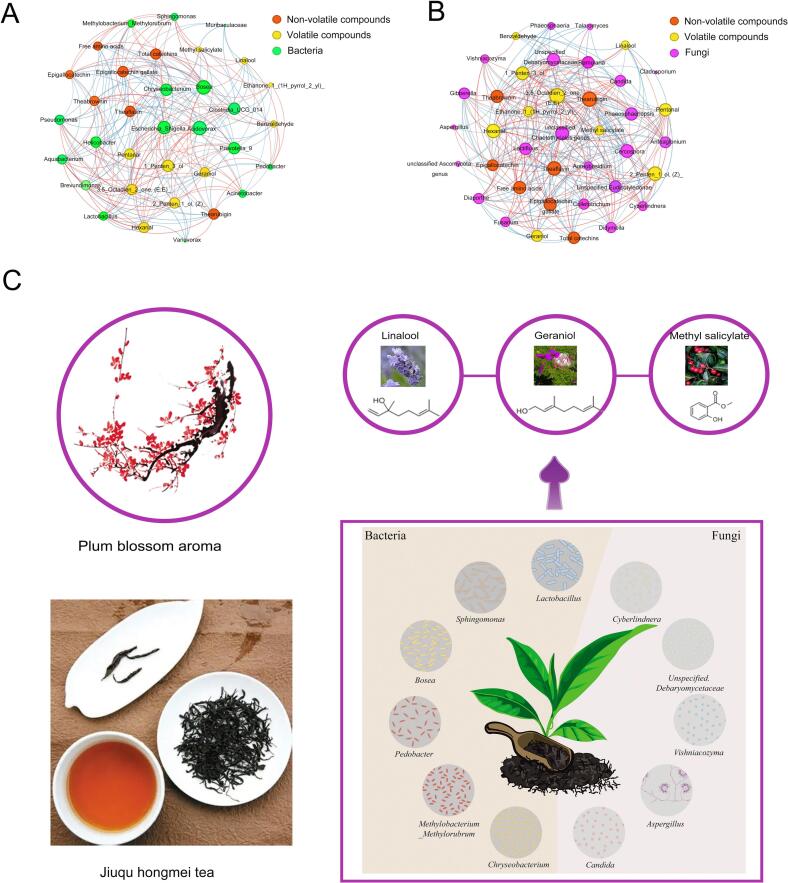
Microbial-metabolite interaction networks and key aroma component analysis of plum blossom characteristic aroma in JQHM tea. (A) Interaction networks between bacteria and non-volatile/volatile metabolites. (B) Interaction networks between fungi and non-volatile/volatile metabolites. (C) Microbial metabolic associations with plum blossom characteristic aroma compounds and core aroma components. Red lines indicate positive correlations, while blue lines indicate negative correlations. Line thickness represents the strength of correlation, and node size reflects the degree of connectivity. (For interpretation of the references to color in this figure legend, the reader is referred to the web version of this article.)

Finally, we constructed a microbial-flavor metabolite visualization network using Spearman correlation analysis (with *r* > 0.7 and *P* < 0.05 as thresholds) to elucidate their underlying associative logic (Ma et al., 2023). In high-grade teas, beneficial bacteria (e.g., *Lactobacillus*, *Sphingomonas*) were strongly positively correlated with positive metabolites (e.g., linalool, EGCG). Notably, *Methylobacterium-Methylorubrum*, a keystone taxon in premium samples, likely drives the synthesis of linalool and geraniol through the shikimate pathway and terpenoid biosynthesis, mediated by specific enzymatic activities such as alcohol dehydrogenases and terpene synthases (Li et al., 2018). Furthermore, these beneficial bacteria exhibited significant negative correlations with potential pathogens found in low-grade teas, indicating ecological regulation via competition (Fig. 4A). Additionally, key fungi such as *Candida* correlated positively with pleasant aroma compounds (e.g., isoamyl acetate) but negatively with off-flavors, highlighting their role in fine-tuning the flavor profile through substrate competition (Fig. 4B). Concurrent with this, *Aspergillus* species contributed to flavor formation by secreting glycoside hydrolases (e.g., β-glucosidases) to release free volatiles, and participating in the biosynthetic routes for salicylic acid derivatives (Yang et al., 2023). This contrast between microbial promotion and inhibition, supported by these specific metabolic routes, offers a mechanistic framework for JQHM quality control.

In summary, as shown in Fig. 4C, the distinctive plum blossom aroma characteristic of high-grade JQHM black tea results from a synergistic interaction between a specialized microbial community and three key volatile compounds. The formation of this aroma follows a coordinated sequence in which microbial metabolic activity drives the synthesis of aroma precursors, leading to the generation of volatile compounds that ultimately define the tea's sensory characteristics. For instance, in the tea fermentation process, different bacterial genera play distinct yet complementary roles. Specifically, *Sphingomonas* modulates carbon–nitrogen metabolism to supply terpenoid precursors; *Methylobacterium–Methylorubrum* optimizes the metabolic environment for aroma precursor generation and potentially regulates the plant's intrinsic biosynthetic pathways; and *Lactobacillus* secretes enzymes that facilitate the transformation of small molecules. Together, these actions collectively lay the foundation for the biosynthesis of key aroma compounds such as geraniol and linalool (Wang, Jia, et al., 2024). Among the fungal community, *Cyberlindnera* contributes to linalool production through the shikimate pathway (Priebe et al., 2021). *Aspergillus* mediates glycosylation modifications (Rivas et al., 2013). Sensorially, linalool provides subtle freshness due to its low odor threshold, geraniol adds sweet, fruity notes, and methyl salicylate introduces a cooling sensation that balances sweetness and extends the aftertaste (Rivas et al., 2013). Together, these traits define the signature “smooth, sweet, and fresh” profile emblematic of high-grade JQHM.

Sensorially, these three volatile compounds exhibit complementary traits: linalool imparts a subtle freshness owing to its low odor threshold, geraniol enhances the aromatic profile with sweet, fruity notes, and methyl salicylate introduces a cooling sensation that balances sweetness and prolongs the aftertaste. Together, they create the signature “smooth, sweet, and fresh” taste and “floral–fruity” aroma emblematic of high-grade black tea. These well-established correlations not only underscore the uniqueness of JQHM among premium black teas but also provide clear targets for future research into the molecular mechanisms of microbial regulation and characteristic aroma formation.

## 4 Conclusion

This study employed an integrated metabolomics and microbiome approach to elucidate the formation mechanism of the characteristic ‘plum blossom’ aroma in JQHM. Our findings establish a direct, causative link between specific microbial taxa and aroma profiles. Premium grades were defined by an enrichment of beneficial microbiota, particularly *Methylobacterium*-*Methylorubrum* and *Aspergillus* species, which drive the biosynthesis of signature compounds, namely linalool, geraniol, and methyl salicylate, via terpenoid pathways and glycoside hydrolysis. Conversely, lower grades exhibited microbial dysregulation, where spoilage microbes accelerated catechin oxidation, leading to elevated TBs and sensory deterioration. The novelty of this work lies in proposing a mechanistic framework for quality control, shifting from mere correlation to identifying specific microbial drivers. This multi-omics association model provides a theoretical basis for precision fermentation and offers a valuable paradigm for flavor modulation in traditional fermented foods.

Despite these advances, functional validation is required to confirm causality. Specific experimental designs should include targeted gene knockout studies to silence key biosynthetic genes such as terpene synthases or glycoside hydrolases, constructing axenic or synthetic co-culture systems to observe interspecies interactions, and employing DNA-based Stable Isotope Probing to trace the metabolic fate of carbon sources into aroma compounds. Furthermore, to resolve the temporal kinetics of flavor formation, future investigations should implement high-resolution time-series sampling throughout fermentation, integrating shotgun metagenomics with meta transcriptomics to elucidate actively expressed metabolic pathways.

## CRediT authorship contribution statement

**Jiang Cao:** Writing – review & editing, Writing – original draft, Visualization, Validation, Methodology, Investigation, Formal analysis, Data curation. **Yiwei Jin:** Writing – original draft, Validation, Formal analysis, Data curation. **Lei Zhang:** Writing – review & editing, Validation, Supervision, Software, Resources, Project administration, Investigation, Funding acquisition, Conceptualization.

## Declaration of competing interest

The authors declare that they have no known competing financial interests or personal relationships that could have appeared to influence the work reported in this paper.

## Data Availability

Data will be made available on request.
