# Supplementary material for: Integrating metabolomics and microbiome analysis to unravel the plum blossom signature of premium Jiuqu Hongmei tea
Source: Food Chem X. 2026 Jun 8;37:104072. doi: 10.1016/j.fochx.2026.104072 (PMC13276576; doi:10.1016/j.fochx.2026.104072)
Supplement: Supplementary file 1 — Supplementary material 1 [file mmc1.docx]

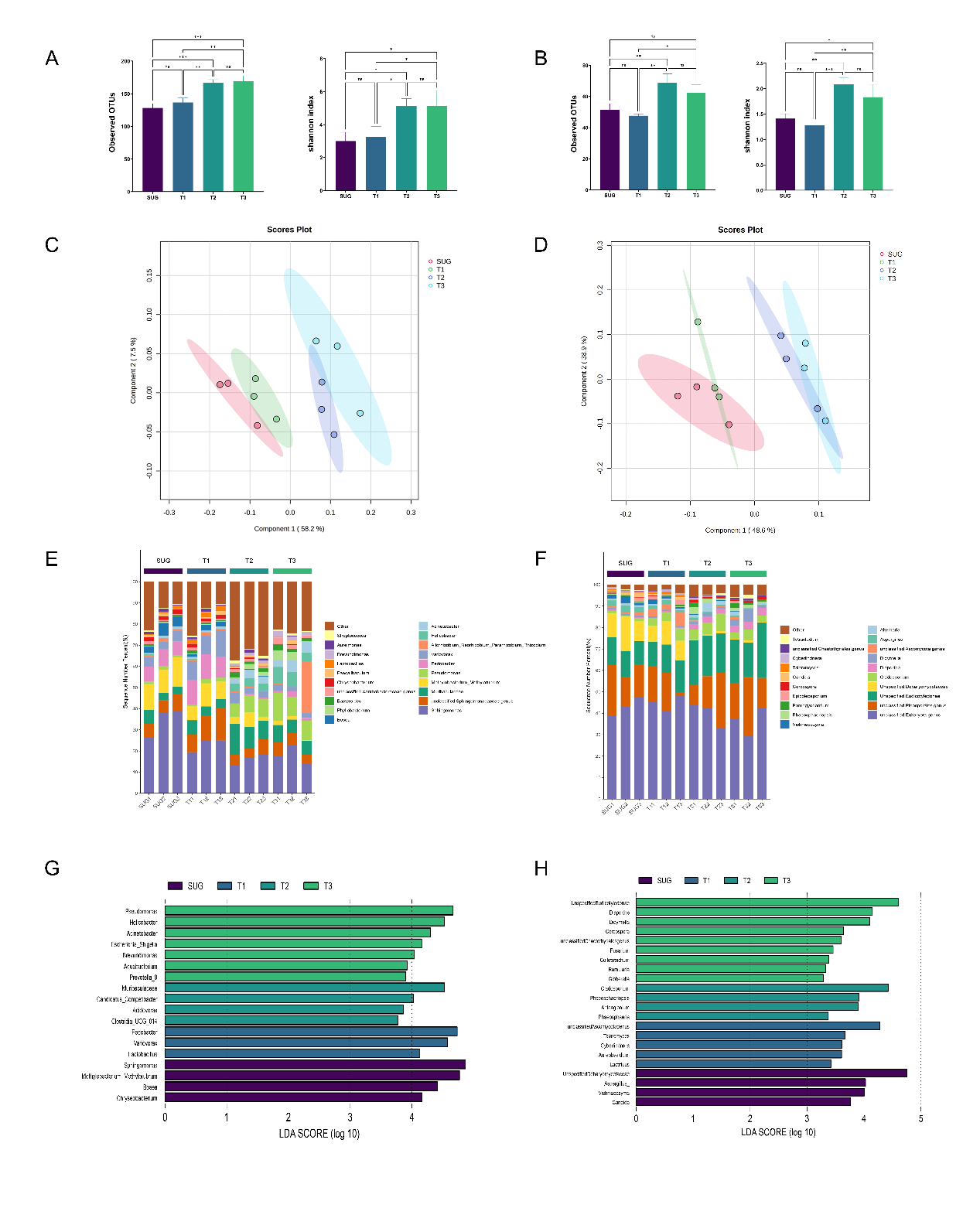


Fig. S1. Microbial analysis of JQHM black tea with different grades. (A) Bacterial α-diversity analysis. (B) Fungal α-diversity analysis. (C) Bacterial β-diversity analysis. (D) Fungal β-diversity analysis. (E) Dominant bacterial genus analysis at genus level. (F) Dominant fungal genus analysis at genus level. (G) LDA scores of bacterial genera across different tea grades. (H) LDA scores of fungal genera across different tea grades. (*n* = 3, ns *P* *>* 0.05, * *P* *<* 0.05, ** *P* *<* 0.01, *** *P* *<* 0.001, **** *P* *<* 0.0001).
